# Between the convention and conventional practice: Israeli social workers' recommendations regarding the legal capacity of people with disabilities

**DOI:** 10.1111/jar.12986

**Published:** 2022-02-10

**Authors:** Roni Holler, Shirli Werner

**Affiliations:** ^1^ Paul Baerwald School of Social Work & Social Welfare The Hebrew University of Jerusalem Jerusalem Israel

**Keywords:** convention on the rights of persons with disabilities (CRPD), guardianship, Israel, legal capacity, supported decision‐making (SDM)

## Abstract

**Background:**

Following the convention on the rights of persons with disabilities (CRPD), various countries have recently amended their legal capacity laws with the aim of restricting the use of guardianship and increasing the use of other, less restrictive practices, mainly supported decision making. As social workers have a key role in carrying out these reforms, this study examines how Israeli social workers make legal capacity‐related decisions.

**Method:**

Semi‐structured interviews with 27 Israeli social workers.

**Results:**

Thematic analysis identified three factors driving social workers' recommendations regarding guardianship and supported decision‐making: the person's diagnosis and functioning level, and the presence of a supportive family. A fourth factor, the person's preferences, played a complex and more limited role.

**Conclusions:**

Many changes have yet to be made to fully apply the support paradigm in Israel, since social workers still tend to base their recommendations on factors not fully aligned with the CRPD.

## 1 INTRODUCTION

Legal capacity is a fundamental right in a liberal democratic society—the building block upon which other rights are formed and realised. It may be construed as the ‘right to have rights’ (Bach, 2017) as it enables people to make decisions and to have those decisions respected (Arstein‐Kerslake, 2017). Historically, however, many social groups have been denied legal capacity, as in the case of women's suffrage until the early 20th century. Another example is people with disabilities, whose legal capacity continues to be denied in contemporary times. This denial is evident in diverse jurisdictions around the globe, from Australia (Watson et al., 2020) and Germany (Fallon‐Kund & Bickenbach, 2017; Müller, 2018) to Ethiopia (Marishet, 2017) and Ghana (Combrinck & Chilemba, 2021). Further, it has penetrated into various decision‐making spheres and life stages, such as marriage, reproductive choices, legal contracts, medical treatments, ageing and end of life care (Arstein‐Kerslake, 2021; Bloomer et al., 2019; Marishet, 2017; Quinn et al., 2018; Watson et al., 2019). The denial of legal capacity in all these cases share a common ground: the notion that individuals in these groups lack the appropriate level of decision‐making skills, also known as mental capacity (Fiala‐Butora & Stein, 2016).

### 1.1 Guardianship and the new alternative of supported decision making

Guardianship constitutes one of the most profound forms of legal capacity denial for people with disabilities. Founded on the principle of *parens patriae* (Dinerstein et al., 2015), under guardianship people are given decision‐making powers over others deemed as lacking mental capacity. Their appointment is often for plenary guardianship, in which they are responsible for making decision in most domains of life (Doron, 2004).

The practice of guardianship, particularly for people with disabilities, has recently come under severe criticism (Arstein‐Kerslake, 2017, 2021; Bach & Kerzner, 2014; Quinn et al., 2018). Drawing on a socio‐critical perspective of disability, the critics have argued that it violates people's autonomy and personhood, is based upon non‐realistic and exclusionary understanding of autonomy, and overlooks less restrictive alternatives, most and foremost supported decision‐making (SDM). Based upon the relational notion of autonomy, which views decision‐making as an interdependent process, SDM is designed to enable people with disabilities retain their legal capacity, while providing them with the necessary support to make decisions. In practice, it involves the appointment of trustees to help people with disabilities make or implement decisions, thus ensuring they have control over how they are supported and by whom, and over the final decision (Series & Nilsson, 2018).

These critiques are the cornerstone of Article 12 of the Convention on the Rights of Persons with Disabilities (CRPD), which calls upon state parties to limit their use of guardianship and develop less restrictive alternatives. Accordingly, many signatories have recently reformed their legal capacity laws with the aim of reducing the use of guardianship and facilitating SDM. Furthermore, although many disability organisations and the UN Committee on the Rights of Persons with Disabilities interpreted Article 12 as calling for the abolition of any guardianship system, in practice reforms in the field tended to take a more conservative view, considering guardianship to be a legitimate, albeit last resort option (De Bhailís & Flynn, 2017).

The Israeli welfare state is a prime example for such reforms. Throughout the years, motivated by humane intentions to protect those deemed mentally incapable, guardianship has become common practice among both the elderly and people with disabilities. A 2016 amendment to the 1962 Legal Capacity and Guardianship Law sought to move the Israeli legal capacity system a major step away from this guardianship regime (Kanter & Tolub, 2017). It adopted the ‘necessity’ and ‘last resort’ principles by stating that guardianship could only be used if necessary to protect the person's interests, rights, and needs, and only after considering other, less restrictive alternatives, SDM being the most appropriate. These changes were of prime importance in inching Israel closer to a ‘support paradigm’ (Series, 2015), in which support is provided to enable people with disabilities to exercise their legal capacity (Holler et al., 2020). Nevertheless, as in other countries, there is still great uncertainty regarding the actual realisation of this reform. This includes, for example, the challenges supporters face and the extent to which their support meets the needs and wishes of people with disabilities (Bigby et al., 2017; Browning et al., 2021). Another significant gap in the literature—and the focus of this study—relates to how social workers make professional decisions under this new regime.

### 1.2 Social work and guardian appointment

In Israel, as elsewhere (Campbell et al., 2018; Dixon et al., 2021), social workers are highly involved in legal capacity proceedings. They are responsible for initiating the appointment process, and for providing the family court with recommendations regarding the measures most appropriate for the specific client they serve as key inputs in the legal outcome (Crampton, 2004; Schindler & Segal‐Reich, 2016(. Despite their cardinal role, hitherto only few studies have examined social workers' practices in the realm of legal capacity (Dixon et al., 2021), and in particular their recommendation process (Barel et al., 2020). Most, moreover, have focused on the elderly, tending to examine social workers' official reports. Although useful for understanding real time recommendations, these reports tend to provide little information on the context of these recommendations and little in‐depth interpretation of their justifications. These few available studies have found that social workers experience a substantial conflict between their human rights values and the need to restrict the legal capacity of their clients (Ruškus et al., 2020); acknowledge the shortcomings of guardianship, but nevertheless consider it vital in protecting disabled individuals from neglect and abuse (Holler & Werner, 2020); and tend to describe their clients' situation mainly in terms of their diagnosis, illness and incapacity (Barel et al., 2020).

In light of the scarce available research in the field, some helpful insights can also be gained from studies on other professionals such as psychologists, psychiatrists, occupational therapists, speech and language therapists, mainly in the context of the UK Mental Capacity Act, 2005. These show that professionals often face challenges in incorporating new legal capacity frameworks; have inaccurate understanding of legal capacity‐related practices, mainly SDM; assess mental capacity based on their clients' perceived best interests; raise concerns over misuse of SDM; are prone to risk aversive interventions; and struggle to provide their clients, particularly those with high communications needs, with adequate support and accommodations (Gooding, 2015; Jayes et al., 2020; Rogers et al., 2020).

Taken together, despite their importance in the new legal capacity landscape, we still know very little on how professionals, mainly social workers, reach their recommendations on guardianship and SDM appointments. The study described in this paper is part of a larger mixed‐methods research project examining social workers' perceptions and practices regarding guardianship and SDM. In a factorial survey study included in this project (Werner & Holler, 2021), we found four client‐level characteristics to be influential in the social workers' recommendation process: diagnosis, level of decision‐making support needed, availability of family support, and client preference. Social workers were more likely to recommend guardianship when the client was diagnosed with intellectual as opposed to mental disability, needed high support in decision making, lacked family support, and agreed to guardianship. The present study aimed to gain deeper insights into the meaning social workers attached to these factors, as well as into their underlying justifications, by analysing their own accounts and experiences.

## 2 METHOD

### 2.1 Participants

To be included, participants had to be licensed social workers specialising in the disability field. Participants were recruited purposively via welfare organisations, professional acquaintance, and social media groups, with the snowball technique employed to recruit additional participants. Twenty‐seven Jewish‐Israeli social workers (23 women) participated in this study (*M*_age_ = 42.3, *SD* = 11.0, range = 24–68). They worked in eight local authorities in different areas in Israel, serving persons with mainly mental and intellectual disabilities—11 as welfare officers; 10 as direct‐care social workers; four as residential care service workers; and two as guardianship agency workers. The participants had an average of 16.1 years' experience social work (*SD* = 9.8 years, range = 3 months‐44 years), and specialised in the disability area for an average of 8.9 years (*SD* = 5.4 years, range 3 months to 17 years).

### 2.2 Procedure

After providing informed consent and having been instructed not to reveal any personal or identifying information on clients, the participants were interviewed in a place of their choosing: work (17), café (4), home (3), the university (2), and community club (1). To compensate for their time while preserving the principle of informed consent, they were given a modest gift voucher worth $30. The interviews were conducted in Hebrew by two trained students with background in social work and experience in qualitative interviewing.

The interviewers asked the participants to elaborate on the meaning of guardianship and SDM and their process of formulating recommendations, including factors considered in the process and their justification. After each of the first interviews, the team discussed the interview and adjusted the interview guide. Thus, for example, to gain deeper understanding of how social workers identified their clients' wishes, we added questions on the setting of the meeting and on the ways these wishes were clarified. Similarly, since it became evident in the first interviews that social workers took for granted the need to appoint guardians for people with intellectual disabilities, we added a follow‐up question to clarify their justification for this. Finally, to elicit real‐life processes and dilemmas, the interviewees were prompted to describe concrete cases.

Each interview lasted 35–95 min. The interviews were audiotaped and transcribed, with all identifying information removed. The Ethics Committee of the Paul Baerwald School of Social work (The Hebrew University of Jerusalem) approved this study.

### 2.3 Data analysis

Data were analysed by developing themes using inductive open coding (Braun & Clarke, 2006). In the first stage, all interview transcripts were read by the two authors and two research assistants to encode units of meaning and make initial notes. The team discussed each of the first 18 interviews jointly in order to agree on the coding system and to aggregate codes with similar meanings into themes. Next, the nine remaining interviews were coded separately by the two authors and at least one research assistant, based on the emerging thematic map. The team members continued discussing their coding and its match with the new data. Throughout this process MAXQDA software was used.

### 2.4 Trustworthiness

To ensure trustworthiness, interview excerpts were presented (using pseudonyms) and an audit trail of the analytic process maintained. Quotes presented below were professionally translated at the manuscript writing stage, with all translation closely reviewed by both authors. Finally, peer debriefing was held in various stages of the data analysis in order to ensure systematic analysis and to verify that each theme and subtheme had a coherent pattern (Nowell et al., 2017).

## 3 RESULTS

Three key factors were found to be at the base of social workers' recommendations regarding guardianship and SDM: the person's diagnosis and decision‐making capacity, and the presence of a supportive family. The person's will plays a more limited and complex role.

### 3.1 ‘Square peg and a round hole’: Diagnosis

The first and key factor underlying social workers' decision‐making is the client's diagnosis. The overwhelming majority of participants perceived guardianship as extremely valuable for people with intellectual disability. In many of these cases, especially when moderate to severe intellectual disabilities were involved, appointing a guardian was conceived as a mandatory and almost default solution. According to Sarit, a welfare officer working with persons with intellectual disabilities and elderly people, ‘from what I've seen, a person diagnosed with intellectual disability needs a guardian in 99.9% of cases’. In fact, while social workers frequently debated on who should be the named guardian, they rarely questioned the need for one in the first place.

Although people with intellectual disabilities were generally perceived as requiring guardianship, their perceived level of disability also played a decisive role. All participants working directly with people with intellectual disabilities perceived clients diagnosed with moderate to severe disability by the Ministry of Welfare as requiring guardianship. Accordingly, although Adi saw SDM as ‘an approach that is very humane and very respectful of human dignity and freedom’, she and others did not see this practice as relevant for people diagnosed with moderate or severe intellectual disability: ‘a square peg and a round hole can never fit […]. It's like we're imposing something inappropriate for this level of functioning, so why?’

Some of the participants also stressed that in recent years, mainly due to the 2016 amendment and the growing rights discourse, the guardianship appointment process has become somewhat less automatic. For them, the need to appoint a guardian for people with intellectual disabilities should be examined on a case‐by‐case basis. According to Dalia, the traditional approach ‘has recently changed […] because there are some cases, not many unfortunately, of people with intellectual disability who don't need a guardian. Support is possible, SDM, and a guardian can be appointed for only some of the [relevant decision‐making] areas’.

### 3.2 ‘Can you count change?’: Decision‐making capacity

In cases considered by the participants as less clear‐cut, they argued that each should be examined individually. In these cases, several additional factors were considered, key among them being the person's perceived decision‐making capacity, such that SDM or no appointment at all were deemed sufficient for those with higher decision‐making capacity. However, there was considerable variability in the way social workers defined and assessed capacity. A key distinction was between the ability to understand the decision‐making process and the ability to make wise decisions. As for the former, many participants equated decision‐making capacity with the person's ability to understand the nature of the dilemma, to weigh relevant information and to reach logical conclusions. Accordingly, not only were the actual decisions made deemed important, but also the individuals' awareness of and ability to justify them:

[…] people that don't understand health check‐ups, you can't explain to them what is happening, what is an MRI […] what medications you need […]. They don't understand their situation and can't make decisions, so it's the guardian's role to decide […] (Sarit).

In assessing their clients' ability to make rational decisions, the participants relied heavily on the individuals' current and past decision‐making experience. Dori, for example, tries to see whether the client ‘understands certain concepts, money. I ask him, “You go to the store? They give you money? Can you count change?”’ Thus, the social workers evinced a static notion of capacity, assuming current and past capacity would remain unchanged. More importantly, they tended to examine whether the person was able to decide independently, overlooking the possibility that this ability could improve given appropriate support.

With regard to the ability to make wise decisions, that is, decisions leading to a socially desirable outcome, the participants recommended SDM or no appointment at all when the person was trusted to make the ‘right’ decision:

I nominated a decision supporter for a client twice, but it was clear to me that he needed help and will take the advice provided by the supporter, but also that they can decide for themselves eventually (Rachel).

Several participants recognised the difficulties involved in this ‘wisdom’ criterion, which is largely based on personal values and norms. Some addressed the fact that we may all make bad decisions or lead different lifestyles without our legal capacity being questioned. In resolving this tension, some related to the level of risk involved in decisions deemed unwise. Sima, for example, explained that ‘If someone buys shoes for 2000 NIS [approximately $600] we will say she is crazy’, and added: ‘She can't give her daughter 2500 NIS and owe money to loan sharks because she needs to buy food. This decision damages her beyond a reasonable degree, because it is not a one‐time slip – she makes this mistake every month’.

Crucially, the two notions—ability to understand the decision‐making process and ability to make wise decisions—were often difficult to untangle. In describing and justifying their assessment process, social workers tended to confound them, usually without recognising the different logics driving them.

### 3.3 ‘Natural protection’: A supportive family

Another factor that played into the interviewees' justifications in situations considered less clear‐cut was the family. When a person was perceived to have a supportive family, appointing a guardian was often seen as less acute:

I had someone who […] until now had two guardians and I saw that overall she was fine, she could take care of herself, she had a caring family that supported her […]. So she did receive my recommendation to take her off guardianship […]. But I must say that this is a rare case (Edna).

Yael provided a description of the other extreme: ‘There are people who have no one to take care of them […] they face a tough reality and it's clear that you're doing the best for them [by appointing a guardian]’.

The availability of a supportive family affects social workers' recommendations in a number of ways. First, under Israeli law, guardians, including family members, are required to report annually to the Administrator General and Official Receiver, enabling the state to monitor the person's financial situation. When the family is perceived as supportive, the risk for exploitation is reduced, making such supervision unnecessary. Second, appointing a guardian is often seen as a way of strengthening the family's commitment to the person in need. Once the family is perceived as committed, this guardianship role becomes redundant. For Neta, some people enjoy ‘natural protection. Even if you won't take any measure, they will be protected’.

Finally, a supportive family is also one in which the person feels comfortable to consult and cooperate, which in turn reduces the fear that the person would make bad decisions. When Rotem was asked in what situations was guardianship mandated, she replied:

[…] more than cognitive ability, it's also the relationship with the parent, how much the child trusts him, listens to him […]. She trusts her parents with money, they have a joint bank account […] so it doesn't bother me very much that there's no guardian.

### 3.4 ‘The child sat and smiled’: Individual preference

A final justification in guardianship and SDM decisions is the individuals' preference. This factor was different from the previous three, as participants rarely raised this justification spontaneously. However, as the guardianship reform calls for eliciting individuals' wishes, we proactively asked the participants regarding their communication with clients during the assessment process. In response to our inquiry, the overwhelming majority suggested the person had a central role. They perceived meeting the clients as enabling the social worker to directly assess their functioning, condition, and family relations. However, the participants hardly referred to the client's preferences. When asked directly, most claimed they were considered their client's preferences, but digging deeper into their accounts revealed a more complex picture.

First, the role of the clients' preference did not usually relate to the appointment itself, but rather to the choice of guardian. Second, it seems the participants usually inquired about the clients' preference only after they had already met the family and decided with them. Thus, the clients were mainly asked to cooperate.

[…] I don't always hold [meetings] with the individual, I meet the family first […] and then we meet together with the family and the individual […]. It's important to me that the expectations are first very clear to the family, and that if they agree to be guardians then we talk about this with the individual. (Adi)

Another complexity emerged when participants tried to clarify the client's preference. In their attempt to simplify the information provided to the individual, they became selective, usually avoiding reference to information on possible alternatives to guardianship. In particular, guardianship itself was frequently presented as advantageous and its absence as harmful. In some cases, guardianship was even presented as a way to continue the supportive relationship with the family and the immediate surrounding. Presented this way, clients naturally prefer the guardianship alternative:

I often phrase the question as ‘Who took care of you until now? Who takes you to school? Who brings you food? Do you want them to continue?’ […] I will always present his opinion in my report. Like, ‘the child sat and smiled’, ‘the young man agreed that the parents would continue to take care of him’. (Michal)

Still another complexity is related to the fact that social workers usually only try to clarify the individuals' preferences in cases of high functioning and verbally communicative clients. Otherwise, they skim through this stage or even skip it:

First, I try to speak to him about daily things […] half of the time I don't even mention the word guardian. For example, I met someone in [a residential setting] who couldn't speak at all […] so there are situations where you don't ask at all. But in other cases you try to simplify things. (Sarit)

## 4 DISCUSSION

Social workers have a key role in carrying out legal capacity reforms given their discretionary power to recommend guardianship or less restrictive alternatives. This study examined how social workers make their recommendations in this new and complex juridical landscape. Our analysis of semi‐structured interviews with Israeli social workers in the disability area yielded four key discretionary factors: diagnosis, decision‐making capacity, supportive family and the clients' preferences. Our results highlighted the complex and diverse ways in which these factors come into play.

One of the criticisms regarding guardianship is that it is often based solely or mainly on the person's *diagnosis*. Our findings echo other critical studies (Jayes et al., 2020) in showing that professionals often recommend guardianship for those diagnosed with intellectual disability almost automatically. In that, they reproduce a practice that has prevailed in Israel for many years. Out of the benign intention of protecting people with intellectual disability, their legal capacity has traditionally been denied with guardianship as the default solution, with little prior reflection or discussion regarding its necessity or appropriateness (Holler et al., 2020; Kanter & Tolub, 2017; Soffer et al., 2017). Our findings show that the legal capacity reform has not yet challenged this substitute decision‐making regime in any substantial way.

Note that the automatic linking of intellectual disability and guardianship, also known as the status approach to legal capacity, runs against the CRPD, which views the former as discretionary on the grounds of disability (Arstein‐Kerslake, 2017). Moreover, this status approach is inconsistent with a growing body of studies demonstrating that decision‐making capacity and related skills are contingent on factors other than cognition (Ganzini et al., 2005; Wehmeyer & Bolding, 2001). These studies reject the view that decision‐making capacity should be determined solely by predefined, individual cognitive skills. These skills are often evaluated without regard for relational factors (Watson et al., 2017), such as support from relatives or paid staff, whereas these studies have shown that given adequate opportunities and support, many people with intellectual disabilities, including those diagnosed as having profound and multiple disabilities, are able to enhance that capacity and decrease the risk involved (Arstein‐Kerslake, 2021; Dukes & McGuire, 2009; Wehmeyer & Bolding, 2001). Similarly and more broadly, it is argued that personhood, including the right to legal capacity, should not be made contingent on an individual's cognitive skills (Kittay, 2019; Watson et al., 2017).

In legal capacity decisions that seem less clear‐cut, mostly with regard to individuals without intellectual disabilities, several additional factors enter into the social workers' consideration, first and foremost perceived decision‐making capacity. Under this functional approach (Series, 2015), that capacity, rather than the impairment, lies at the heart of the assessment. Interestingly, the participants tend to shift between a best interests and a procedural understanding of capacity—the ability to make wise decisions as opposed to the ability to understand the decision‐making process (Owen et al., 2009).

A best interests test of legal capacity, also known as the outcome or substantive approach to legal capacity (Series, 2015), assumes that the removal of legal capacity should be reserved to cases where persons make unwise decisions that place them at risk. This resonates well with Israeli law, which requires the appointment of a guardian if it necessary to protect the person's interests, rights, and needs (Kanter & Tolub, 2017). It also resonates with social workers' tendency for risk aversion and their perceived duty to protect their clients from harm (Werner & Holler, 2020).

However, although protecting people's interests is laudable, disability scholars have rightly pointed out crucial flaws with prioritising it in a legal capacity test. In particular, this runs the risk of denying capacity based on the assessor's own values (Arstein‐Kerslake, 2017), especially when social workers are provided with little specific guidance on how to assess a ‘person's interests, rights, and needs’, with Israeli law being a case in point. Another fundamental flaw with the best interests test is its inherent inequality in being applied primarily to specific segments of the population, particularly people with disabilities (Arstein‐Kerslake, 2017).

Embodied in some jurisdiction, most notably in the UK Mental Capacity Act, 2005 (Donnelly, 2009), procedural assessment is designed to overcome the bias involved in assessing people's decision‐making capacity based on perceived best interests and to avoid denying a person's autonomy based on the assessor's own values. Despite overcoming some of the problems associated with both the diagnostic and substantive understanding of capacity, this approach is also not without its downsides. For one, assessors often fail to separate procedural from substantive capacity, which leads evaluations to ‘smuggle in’ hidden normative judgement about what is normal, valued and desired (Donnelly, 2009; Freyenhagen & O'shea, 2013; Murrell & McCalla, 2016; Series, 2015). This was also the case with our participants.

Importantly, both procedural and substantive approaches to capacity testing tend to view (in)capacity as the property of the individual, overlooking the dynamic social nature of decision‐making and autonomy. For example, our participants based their assessments on their clients' past and current experiences, without considering that many of them had grown in a substitute decision‐making regime, with limited opportunities to experience and develop a decision‐making capacity of their own. More importantly, by focusing on procedural tests as well as on substantive capacity, our participants overlooked the possibility that capacity might be enhanced once decision‐making support is provided (Bach & Kerzner, 2014).

Our findings also point to the importance of *family support* in assessing the need for guardianship. Having a supportive family, in the social workers' view, reduced the need to appoint both a guardian and SDM. This resonates with the relational approach to autonomy, as well as with the traditional social work approach of viewing the person in her environment (Weiss‐Gal, 2008). In particular, it acknowledges that risk and vulnerability are not necessarily inherent but rather contingent upon the availability of a supportive family.

Viewing the family as a valuable source of support is deeply rooted in Israeli culture, which despite post‐industrial individualization processes continues to be highly familailist (Fogiel‐Bijaoui, 2002). In the context of legal capacity, such norms can be highly valuable for supporting people with disabilities. Importantly, however, such focus on the family might overlook the fact that non‐familial supporters such as paid professionals or volunteers can provide SDM. Israeli Law recognises these alternative sources of support and recently, the authorities have begun providing training courses for professional supporters. Our findings suggest that social workers are still somewhat behind in recognising the legitimacy of these sources of support. In addition, we should not lose sight that the family was often viewed by our interviewees as responsible for directing the person to make ‘wise’ decisions. Such a view, which incorporates a substantive notion of mental capacity, runs the risk of misinterpreting the rationale of the relational approach to legal capacity: supporting people in making their own decisions.

Finally, an important criticism against guardianship appointment processes is the lack of reference to the person's *preferences*. Studies show that they are not at all presented at court (Doron, 2004; McSwiggan et al., 2016), conversely, a recent Israeli study showed that they are duly reported in social workers' reports (Barel, 2018). This gap may lie in the fact that the social workers serve in practice as ‘court visitors’, in charge of informing the court of their clients' wishes (Crampton, 2004). Our findings indicate that this task is far from streightforward and when carried out, often diverges significantly from its stated objective: to authentically understand the person's preferences. This may be due to several reasons, including the way the issue is presented to the person and the lack of recognition of the ability of people communicating non‐verbally to understand the issue and express their will.

Importantly, the assessment is usually made by a social worker only minimally acquainted with the person. Such limited acquaintance may pose as a barrier as studies, including those focused on people with profound and multiple disabilities, have shown that when choices are communicated to them in meaningful ways by people familiar to them, they often have unexpected abilities to make decisions (Vehmas & Mietola, 2021). Moreover, studies show that having close relationship and personal knowledge about the person is often necessary to reveal and give meaning to nonverbal forms of communication (Watson et al., 2017).

### 4.1 Limitations

Two research limitations should be taken into consideration. First, the generalizability of our findings might be limited due to being based on a convenience sample of Jewish social workers. Note, however, that our sample was comprised of professionals with various levels of experience, working with different populations and in different settings and geographical areas. Second, due the ideological and highly controversial issues raised in the interviews, social desirability bias should also be considered.

### 4.2 Implications for practice

Our findings suggest some practical implications. Most importantly, comprehensive professional guidelines must be developed. These should discuss factors that social workers should and should not take into consideration in their recommendation process, as well as outline the way social workers should identify and address clients' preferences. In order to be more closely aligned with the support paradigm (Series, 2015), these guidelines should emphasise the relational nature of autonomy, which stipulates that autonomy ‘requires interdependence, not isolated independence’ (Dowling et al., 2019, p. 1059). In practice, this means encouraging social workers to ask not whether a certain individual is able to decide, but what tools are available for that individual to do so. Relatedly, given that the guardianship appointment process involves normative judgement and can dramatically change people's lives, we share Freyenhagen and O'shea (2013) recommendation of increasing the transparency and accountability of the process and its guidelines. Lastly, to put these guidelines to use, comprehensive training should be provided to social workers including familiarity with the CRPD and Israeli law, and in‐depth discussion on the professional and ethical dilemmas involved.

## 5 CONCLUSION

Although Israel has taken important steps towards a support paradigm within its reformed law, many changes still have to be made in order to make this paradigm a reality. Social workers still tend to base their recommendations on factors not fully aligned with the CRPD, such that many clients remain subjected to guardianship that deprives them de facto of their legal capacity.

## Data Availability

Given the delicate nature of the qualitative data in this study, and due to strict ethical guidelines regarding our participant's privacy, the data for this study cannot be made publicly available. However, readers are encouraged to contact the authors with any questions. Wherever possible we will share specific information with the reader.
